# The WASP-Arp2/3 complex signal cascade is involved in actin-dependent sperm nuclei migration during double fertilization in tobacco and maize

**DOI:** 10.1038/srep43161

**Published:** 2017-02-22

**Authors:** Xiongbo Peng, Tingting Yan, Mengxiang Sun

**Affiliations:** 1 State Key Laboratory for Hybrid Rice, College of Life Sciences, Wuhan University, Wuhan 430072, China

## Abstract

Sperm nuclear migration during fertilization in *Arabidopsis* and rice has recently been found to be actin-dependent, but the driving force behind this actin cytoskeleton-dependent motion is unclear. Here, we confirmed that the actin-dependent sperm nuclei migration during fertilization is a conserved mechanism in plants. Using *in vitro* fertilization systems, we showed that a functional actin is also essential in maize and tobacco for sperm nuclei migration after gamete membrane fusion. Cytoskeleton depolymerization inhibitor treatments supported the view that sperm nuclei migration is actin-dependent but microtubule-independent in both egg cell and central cell during double fertilization. We further revealed that the actin-based motor myosin is not the driving force for sperm nuclear migration in maize and tobacco. The WASP-Arp2/3 complex signal cascade is shown here to be involved in the regulation of sperm nuclear migration in maize and tobacco. It is interesting that sperm nuclei migration within somatic cell also need WASP-Arp2/3 complex signal cascade and actin, suggesting that the mechanism of sperm nuclear migration is not gamete specific.

The double fertilization process that occurs in flowering plants was discovered a century ago^1^,^2^. In this process, one of the two sperm cells from one pollen tube fuses with the egg cell to produce a zygote that then develops into an embryo. The other sperm cell fuses with the central cell to initiate the development of endosperm. The cytology of gametes and the timeline of fertilization process have been well described^3^,^4^,^5^, while most of the very early cellular events just after membrane fusion and molecular mechanisms involved remains relatively unknown^6^,^7^,^8^,^9^. This is largely due to double fertilization occurs internally, deep within maternal tissues and is consequently inaccessible experimentally.

The techniques for *in vitro* fertilization, which are known to be effective in the study of fertilization in animals and lower plants, put a light on the study of early events during and after fertilization in higher plants^10^. *In vitro* fertilization systems were established in maize, tobacco, white and rice^11^,^12^,^13^,^14^,^15^,^16^,^17^,^18^. Based on these systems, some important events during fertilization, such as differential fertilization, egg cell activation, block to polyspermy, have been investigated^19^,^20^,^21^,^22^,^23^,^24^,^25^. Another very important event during fertilization is integration of the male nucleus into each female gamete to fulfill karyogamy. The processes from membrane fusion to nuclear fusion have been investigated^26^. However, the cellular mechanisms underlying the migration of male nucleus and female nucleus in angiosperms had not been elucidated until recently^9^,^27^,^28^.

In animals, pronuclear migration relies mainly on microtubules. In animals, such as sea urchins^29^, *Caenorhabditis elegans*^30^, rabbits^31^, sheep^32^, cows^33^ and pigs^34^, the female pronucleus appears to migrate toward the male pronucleus along microtubules of the sperm aster, which is nucleated from the centrosomes associated with the male pronucleus and appears responsible for pushing the male pronucleus centripetally^35^.

It is noteworthy that, in contrast with animal cells, a number of subcellular organelles in plants have been shown to be associated with actin but not microtubules for their intracellular movement^36^,^37^. Recent experiments in rice also showed that movement of sperm nuclei adjacent to the egg nucleus occurs in an actin-dependent manner^27^,^28^. It was also reported that microtubules are dispensable for migration of male and female gamete nuclei of *Arabidopsis*, while dynamic F-actin activated by ROP8 (Rho-GTPases 8) is necessary^9^. However, the driving force behind this actin cytoskeleton-dependent motion of sperm nucleus is unclear. In addition, whether the actin-dependent movement of sperm nucleus is gamete-specific in higher plants is an interesting question and thus is worthy to be further investigated.

We examined whether actin or microtubules are responsible for the migration of sperm nucleus and female gamete nuclei in tobacco and maize. The results indicated that the sperm nuclear migration is actin- but not microtubule-dependent. Moreover, WASP-Arp2/3 complex signal cascade but not myosin motors are involved in regulating sperm nuclear migration in maize and tobacco. This mechanism does not seem to be gamete specific, because sperm nuclear migrated in protoplast also depends on WASP-Arp2/3 complex signal cascade.

## Material and Methods

### Plants

Plants of *Nicotiana tabacum* were grown in a greenhouse under 16 h photoperiod at 25 °C. Maize (*Zea mays* L.) plants of inbred lines A 188 were grown in a greenhouse under 16 h photoperiod at 25 °C. The ears of the maize plants were bagged before silk emergence to prevent pollination. Ears were collected at a fertilization receptive stage when emerged silks reached 12–13 cm in length.

### Isolation of female cells, sperm cells and ovule protoplast

Egg cells of maize were isolated from ovules dissected from mature ears of maize as previously described by Peng *et al*.^13^. Half-ovules were incubated for 5 min at room temperature in an enzymatic solution containing 0.5% Macerozyme Onozuka R10 (Yakult Honsha, Tokyo, Japan), 0.5% Cellulase Onozuka R10 (Yakult Honsha, Tokyo, Japan), 3 mM 2-(N-morpholino)-ethanesulfonic acid (MES), adjusted to 725 mOsmol/kg H_2_O with mannitol, pH 5.7. Ovule pieces were then washed with 12% mannitol and egg cells were gently picked out from the embryo sac by manual microdissection under an inverted microscope (Olympus CK-30).

Sperm cells of maize were released from freshly collected pollen grains after an osmotic shock in 12% mannitol, and isolated as previously described by Kranz *et al*.^17^.

Female gametes of tobacco were isolated from ovules dissected from flowers collected one day after anthesis using a method modified from Sun *et al*.^18^. Ovules were incubated for 3 hours at 30 °C in an enzymatic solution containing: 1.5% cellulase Onozuka R10 and 0.8% macerazyme Onozuka R10 dissolved in MES buffer (3 mM MES obtained from Sigma, 13% mannitol, pH 5.6). Ovules were then washed with MES buffer for three times, and female gametes were isolated using a glass microneedle under an inverted microscope (Olympus CK30).

Sperm cells of tobacco were isolated using a modified method from Sun *et al*.^18^. Stigmas were artificially pollinated 1 day after emasculation. After 35 h of pollen tube growth *in vivo*, pistils were collected and surface-sterilized in 75% ethanol for a few seconds. Pistils were rinsed twice in distilled water and then cut off. The cut end was placed in liquid medium containing 20% sucrose, 0.01% boric acid and 0.1 mM CaCl_2_, and then cultured in the dark at room temperature. After pollen tubes emerged from the cut end, they were placed in 12% mannitol solution, where the tubes burst and sperm cells were released.

For ovule protoplast isolation, half-ovules were incubated for 30 min at room temperature in an enzymatic solution containing 0.5% Macerozyme Onozuka R10 (Yakult Honsha, Tokyo, Japan), 0.5% Cellulase Onozuka R10 (Yakult Honsha, Tokyo, Japan), adjusted to 725 mOsmol/kg H_2_O with mannitol, pH 5.7. Ovule pieces were then washed with 12% mannitol and ovule protoplasts were gently picked out from ovule pieces by manual microdissection under an inverted microscope (Olympus CK-30).

### Cell fusion

The fusion of one sperm cell with one egg cell or one ovule protoplast of maize was performed in BSA fusion medium containing 3 mM MES, 0.1% (W/V) bovine serum albumin (BSA). and adjusted to 750 mOsmol/kg H_2_O with mannitol, pH 6.0 as previously described^13^.

The fusion of an egg cell or central cell and a sperm cell pair of tobacco was performed using a method modified from Sun *et al*.^18^. The fusion was performed in polyethylene glycol (PEG) medium containing 5 mM calcium, 3 mM MES, 20% (W/V) PEG and 5% (W/V) mannitol, pH 6.0.

### Drug Treatment

Two actin-perturbing drugs, cytochalasin B (CB, Sigma) and latrunculin A (LatA, Molecular Probes) were used. Stock solutions of CB (stock 20 mM. working concentration 20 μM) and LatA (stock 1 mM, working concentration 1 μM) were prepared in dimethylsulphoxide (DMSO, Sigma).

The microtubule inhibitor colchicine (Sigma, working concentration 2 mM) and myosin inhibitor 2,3-butanedione monoxime (BDM) (Sigma, working concentration 2 mM or 20 mM) were freshly dissolved to the required concentration in solution just prior to the experiment.

The N-WASP inhibitor wiskostatin (Calbiochem) was used. A stock solution of wiskostatin (5 mM) was prepared in dimethylsulphoxide (DMSO, Sigma). It was freshly diluted to 5 μM concentration in BSA solution or Ca-Mg solution just prior to the experiment.

### Nuclear staining using DAPI

Isolated sperm cells of maize were stained with 4,6-diamidino-2-phenylindole (DAPI, Sigma) and later used for *in vitro* fertilization. Sperm cells of maize were incubated with 2 μg/ml DAPI for 20 min. They were then washed twice in BSA solution and used for fusion. Artificial zygotes or primary endosperms of tobacco were stained with DAPI (0.5 μg/ml for 5 min) after fusion.

### Microscopy and image processing

To visualize the process of sperm nuclear migration and fluorescence of DAPI-stained nuclei, cells were examined using a Leica epifluorescence inverted microscope with a ×63 objective, equipped with a cooled CCD (RTE/ CCD-1300-Y/HS, Roper Scientific Inc.). Digital images from CCD were captured on a computer equipped with the MetaMorph software (Universal Imaging Corporation Inc.).

To visualize the movement of mitochondria in tobacco, the tobacco plants expressing a mitochondrial marker, *p35S::coxlV-GFP*, were used. Tobacco leaves were observed by confocal laser scanning microscopy on a TCS SP2 system (Leica). The 488 nm line of an argon/krypton laser was used for excitation. The fluorescence light longpass was filtered from 515 to 550 nm before being detected by a photomultiplier.

## Results

### Sperm nuclear migration to the nucleus of egg cell and central cell

**Figure 1:**
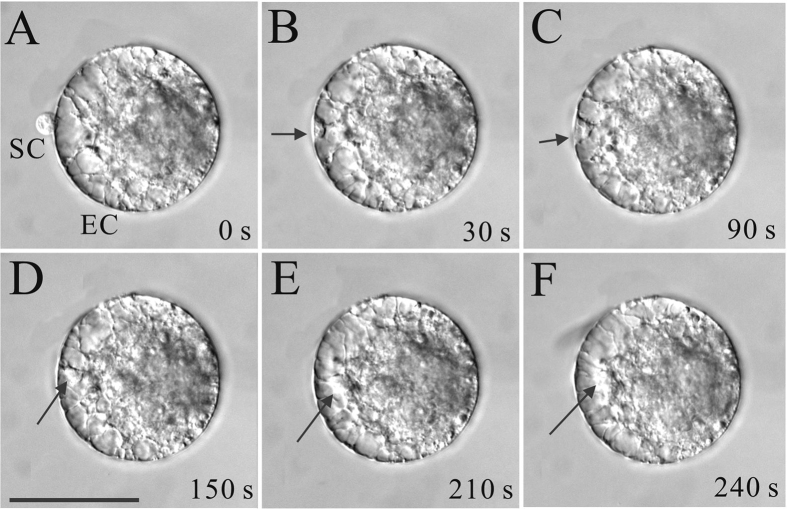
Time-lapse images of the sperm nuclear migration process after *in vitro* fusion of the maize sperm cell (SC) and egg cell (EC) induced by 0.1% BSA medium. Images were taken at varying time intervals over a period of 240 s, as indicated in the bottom right corner of each image. The arrows indicate the sperm nucleus and arrowheads indicate cytoplasmic strands. Scale bar = 50 μm.

In maize, after plasma membrane fusion, the sperm nucleus began to leave the fusion site within a mean time of 10–30 s. Cytoplasmic strands appeared to connect the egg nucleus to sperm nucleus. Then the sperm nucleus migrated from entry point to egg nucleus with a mean velocity of about 3 μm/min. The time course and dynamics of sperm nuclear migration are showed in Fig. 1 and supp movie 1.

**Figure 2:**
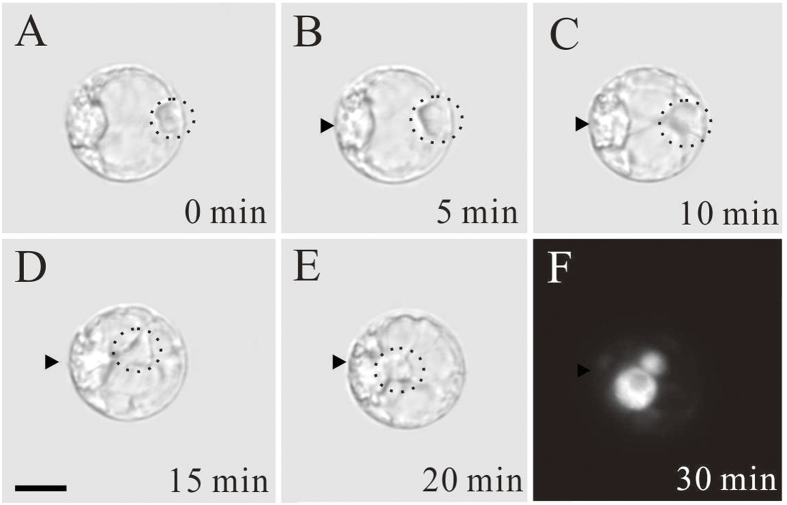
Time-lapse images of the sperm nuclear migration process after *in vitro* fusion of the tobacco sperm cell and egg cell induced in PEG medium. (**A–F**) Images were taken at varying time intervals over a period of 30 min, as indicated in the bottom right corner of each image. Dotted circles indicate sperm nucleus location and arrowheads indicate the nucleus of egg cells in **A–E**. (**F**) The fertilized egg cell shows two nuclei stained by DAPI 30 min after membrane fusion. The smaller structure is the sperm nucleus and the bigger structure is the nucleus of the egg cell. Scale bar = 10 μm.

Due to the presence of cytoplasmic mass around the egg nucleus in maize egg cells, it was difficult to visualize the details of sperm nuclear migration. The egg cell and central cell of tobacco, however, are transparent due to their big central vacuoles. Thus, we visualized the sperm nuclear migration in tobacco. In tobacco egg cells the observations were somehow different compared to those made with the maize egg cell. After fusion the sperm nucleus remained on entry point for about 10 min (Fig. 2A–C). In this time period, some cytoplasmic strands appeared to produce a transvacuolar bridge between the egg nucleus and the sperm nucleus (Fig. 2C). Then the sperm nucleus began to migrate towards the egg nucleus (Fig. 2D–E). The complete time course from the moment the sperm nucleus left the cortical cytoplasm to its arrival at the egg nucleus took 15 min.

**Figure 3:**
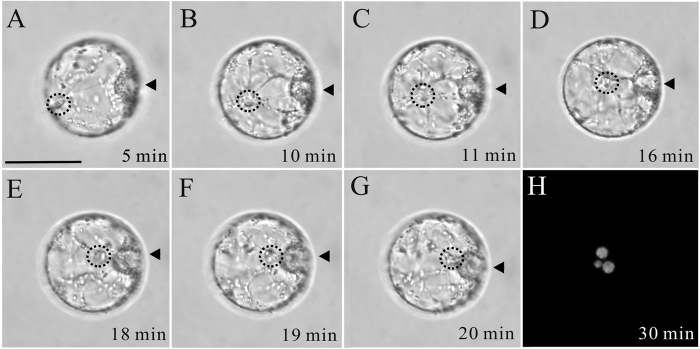
Time-lapse images of the sperm nuclear migration process after *in vitro* fusion of the tobacco sperm cell and central cell induced in PEG medium. (**A–G**) Images were taken at varying time intervals over a period of 30 min, as indicated in the bottom right corner of each image. Dotted circles indicate sperm nuclear location and arrowheads indicate polar nuclei of the central cell in (**A–G**). Cytoplasmic strands around sperm nucleus were also present (**A–G)**. (**H**) The fertilized central cell shows three nuclei stained by DAPI 30 min after membrane fusion. The smallest nucleus is from the sperm, the other two larger nuclei are the polar nuclei of the central cell. Scale bar = 50 μm.

The sperm nuclear migration behavior in tobacco central cells is similar to that of tobacco egg cell (Fig. 3). The entire process from the sperm nucleus leaving cortical cytoplasm to its arrival at the polar nuclei took 20 min. Since the diameter of a central cell is about 50 μm, the velocity of migration of the sperm nucleus was estimated to be about 2.5 μm/min.

### Sperm nuclear migration in female gamete cells depends on actin but not microtubules

**Figure 4:**
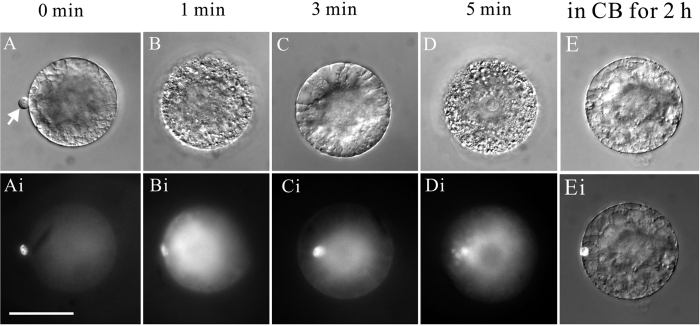
The actin-perturbing drugcytochalasin B inhibits sperm nuclear migration in maize. (**A–D**) Time-lapse images of the sperm nuclear migration process after *in vitro* fusion of the maize sperm cell and egg cell after treatment with the microtubule-depolymerizing drug colchicine. Arrows indicate sperm cell’s contact with the egg cell in **A**. (Ai–Di) DAPI fluorescence images correspond to (**A–D**) show sperm nuclei location at varying time intervals, as indicated above each image. Sperm nuclei were stained by DAPI before fusion. (**E**) Bright field image of a fertilized egg cell kept in medium containing cytochalasin B for 2 hours. (Ei) Bright field and DAPI fluorescence image of the same cell in **E**. The bright white dot indicated the sperm nucleus, which remained at the entry site. Scale bar = 50 μm.

To determine the molecular basis of sperm nuclear migration, we employed a series of drugs that disrupt specific components of the cytoskeleton. Drug inhibitor studies were carried out on maize initially. Prior to fertilization, maize egg cells were treated for 15 min with the microtubule-depolymerizing drug colchicines. Then the fusion of egg cell and sperm cell was induced in BSA solution containing colchicine. The sperm cell could fuse with the egg cell normally, and sperm nucleus could migrate to egg nucleus within 5 min (Fig. 4A–D, Ai–Di), indicating that sperm nuclear migration is not be affected by colchicines (n = 8).

In contrast, prior to fertilization, maize egg cells that were treated for 15 min with the actin-depolymerizing drug cytochalasin B (CB) were affected by the drug. The fusion of egg cell and sperm cell was induced in BSA solution containing CB. Although sperm cells could fuse with egg cells normally, sperm nuclei were unable to begin migration and remained at the entry point (Fig. 4E and Ei, n = 12). Lat A, another actin filament-disrupting drugs, also inhibited migration of sperm nuclei (n = 8).

**Figure 5:**
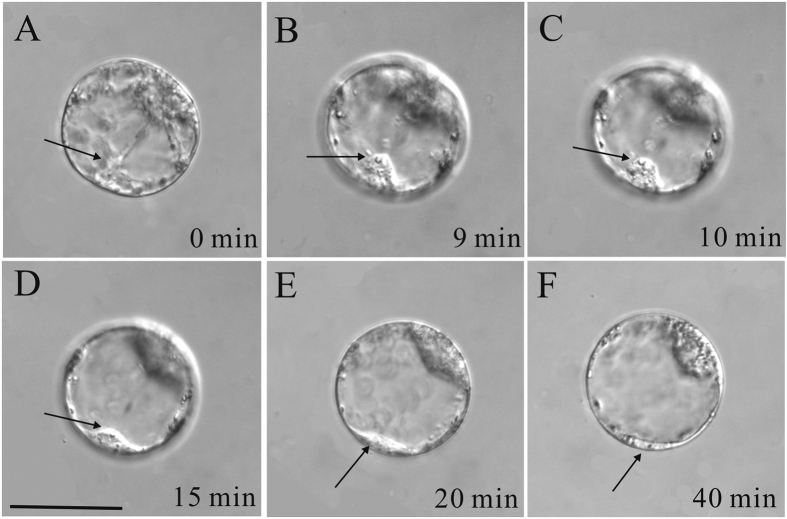
Time-lapse images of cytochalasin B inhibiting sperm nuclear migration in tobacco. (**A**) 10 minutes after *in vitro* fusion of the tobacco sperm cell and central cell, cytochalasin B was added to the medium, when the sperm nucleus was migrating to polar nuclei on the cytoplasmic strands (time 0 minute). (**B–F**) As incubation with the drug proceeded, cytoplasmic strands between sperm and polar nuclei disappeared gradually. Sperm nucleus returned to and remained at the entry site. The arrows indicate the sperm nucleus. Scale bar = 50 μm.

The drug treatments were extended subsequently to tobacco for further analysis. Here we report data mostly from the CB treatment in tobacco fertilized central cells. The fusion product of sperm cell and central cell was treated by CB 15 min after fusion (time 0 minutes). At this time there were cytoplasmic strands between the sperm nucleus entry point and polar nuclei, and sperm nucleus migrated for a distance (Fig. 5A). The cytoplasmic strands were disrupted within 10 min (Fig. 5B,C) and the sperm nuclei went back to fusion point accompanying with the mass of disrupted cytoplasmic strands and remained there (Fig. 5D–F). These results suggested that cytoplasmic strands contain actin, which is essential for sperm nuclear migration.

In tobacco, treatment with the microtubule-depolymerizing drug colchicine did not appear to affect sperm nuclear migration (Table 1).

### Sperm nuclear migration in female gamete cells does not depend on myosin

Observations on artificial zygotes or primary endosperm cells in maize and tobacco demonstrated that sperm nuclear migration clearly depend on the actin cytoskeleton. It is well established that myosins convert chemical energy from ATP hydrolysis into physical movement along actin filaments^38^,^39^,^40^, carrying organelles or RNA/protein complexes with their tails^40^. In an attempt to identify the driving force behind this actin cytoskeleton-dependent motion, the involvement of myosin motors was firstly investigated. The common myosin inhibitor 2,3-butanedione monoxime (BDM), a specific inhibitor of myosin ATPase^41^, was used.

To test the ability of BDM to inhibit myosin activity, we performed experiments using transgenic tobacco expressing a mitochondrial marker, *p35S::coxlV-GFP*^42^. Mitochondria in plants are known to be transported by myosin^43^. In our drug treatment experiments, prior to treatment of *p35S::coxlV-GFP* plant leaf with BDM, mitochondria visualized by coxlV-GFP displayed intracellular movements (supp movie 2). Treatment of *p35S::coxlV-GFP* plant leaf with 2 mM BDM halted mitochondrial movement within 5 min (supp movie 2). Five minutes after BDM had been washed out, mitochondria recovered their movement (supp movie 2). The results suggested that BDM could inhibit myosin activity, and the movement of mitochondria depends on myosin.

In tobacco, even at concentrations as high as 20 mM BDM, sperm nuclear migration could not be inhibited (Table 1). These results indicated that sperm nuclear migration does not depend on myosin.

### Wiskostatin blocks sperm nuclear migration in female gamete cells

We observed that, upon entry into the central cell, cytoplasmic strands between the sperm nucleus and female nuclei were formed, and that the sperm nucleus migrated along these cytoplasmic strands. The cytoplasmic strands were disrupted within 10 min treatment by actin-depolymerizing drug CB. These results suggested the involvement of actin assembly in the process of sperm nuclar migration. In plants, F-actin assembly is regulated by formins and the Arp2/3 complex^44^,^45^,^46^,^47^,^48^,^49^,^50^,^51^. These results led us to perform another experiments to study whether the Arp2/3 complex was involved in the sperm nuclear migration.

**Figure 6:**
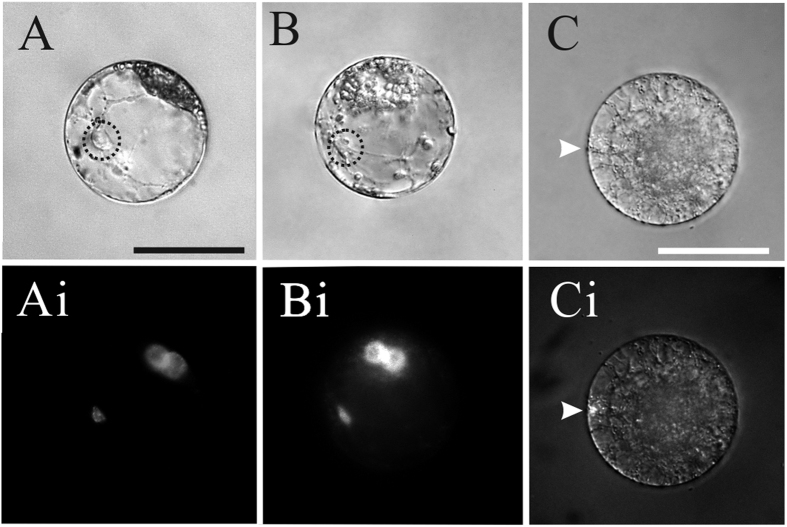
N-WASp inhibitor wiskostatin inhibits sperm nuclear migration in tobacco and maize. (**A**) About 10 minutes after *in vitro* fusion of the tobacco sperm cell with the central cell, wiskostatin was added to the medium. Note at the time of treatmentthe sperm nucleus was in transit to the polar nuclei. (Ai) DAPI fluorescence images of the same cell in **A**. (**B**) 15 minutes after being treated by wiskostatin, the tobacco sperm nucleus remained in the same location but its migration was stopped by wiskostatin treatment. (Bi) DAPI fluorescence images of the same cell in **B**. (**C**) Maize sperm nuclear remained at the entry site of the egg cell treated with wiskostatin. (Ci) DAPI fluorescence images of the same cell in **C**. Scale bar = 50 μm.

The Arp2/3 complex cannot initiate the actin nucleation and polymerization on its own; efficient nucleation of actin polymerization by the Arp2/3 complex depends on the presence of an Arp2/3 activator: Wiskott–Aldrich Syndrome protein (WASP)/suppressor of cAMP receptor (Scar)/WASP family verprolin homologous (WAVE)^52^,^53^,^54^,^55^. Recently, a cell-permeable chemical inhibitor of WASP, wiskostatin, was identified^56^,^57^. Wiskostatin blocks WASP activity by stabilizing its autoinhibited conformation^57^. Wiskostatin was used to determine whether the Arp2/3 complex is involved in sperm nuclear migration. In tobacco, the fusion products of sperm cell and central cell were treated by 5 μM wiskostatin 10 min after fusion, when sperm nucleus had already travelled along the cytoplasmic strand to polar nuclei (Fig. 6A and Ai). Sperm nuclear migration slowed down gradually and reached immobility nearly at the midpoint of the journey, 15 min post- treatment. Transvacuolar cytoplasmic strands between polar nuclei and sperm nucleus remained in the fusion products during the time upon wiskostatin applications (Fig. 6B and Bi). In parallel, we observed the similar result in the case of Maize. Prior to fertilization, egg cells were treated for 10 min with 5 μM wiskostatin. Sperm nucleus remained at the entry point after fusion and was unable to move toward polar nuclei (Fig. 6C and Ci). These results suggested the N-WASP activated Arp2/3 complex mediated actin assembly is involved in sperm nuclear migration in tobacco and maize.

### Sperm nuclear migration in somatic cell is also actin-dependent

**Figure 7:**
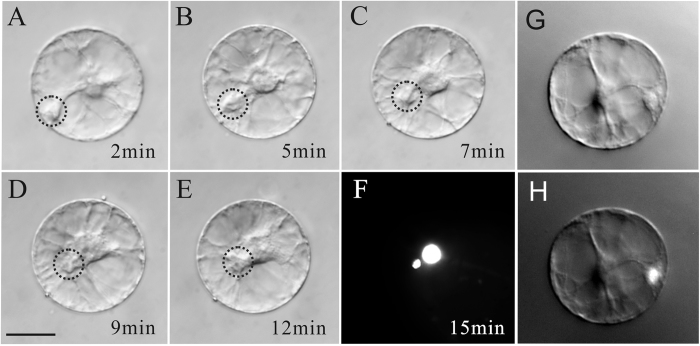
N-WASP inhibitor wiskostatin inhibits sperm nuclear migration in maize ovule protoplasts. (**A–F**) Time-lapse images of the sperm nuclear migration process after *in vitro* fusion of a maize sperm cell with a maize ovule protoplast. Images were taken at varying time intervals over a period of 15 min, as indicated in the bottom right corner of each image. The dotted circles indicate sperm nucleus location in (**A–E)**. (**F**) The fused ovule protoplast shows two nuclei stained by DAPI 15 min after membrane fusion. The small nucleus is the sperm nucleus and the larger structure is the nucleus of the ovule protoplast. (**G**) Maize sperm nucleus remained between the entrance site and the nuclei of ovule protoplast treated with wiskostatin. (**H**) DAPI fluorescence images of the same cell in (**G**). Scale bar = 20 μm.

To investigate whether the mechanism underlying sperm migration is gamete specific, we induced the fusion of sperm cell and ovule protoplast and investigate the mechanism involved in sperm migration in ovule protoplast. After fusion, it was clearly observed that the sperm nucleus could migrate to the nucleus of the ovule protoplast along the cytoplasmic strand. Within about 12–18 min, the sperm nucleus could finally reach the nucleus of the somatic cell (Fig. 7A–F). We also used cytoskeleton inhibitors to treat the fusion product and found that once actin was disrupted, the sperm nucleus movement was also affected, indicating that the process of sperm nuclear migration in somatic cell is actin-dependent (Table 1), similarly to that in egg cells and central cell. Further experiments showed that BDM treatment could not inhibit sperm migration in somatic cells from ovules (Table 1), while wiskostatin treatment inhibited sperm nuclear migration in somatic cells (Fig. 7G–H).

## Discussion

### Migration of sperm nucleus is actin but not microtubule dependent in both monocot and dicot species

In contrast to animal nuclei, which are moved by motor proteins (kinesins and dyneins) along the microtubule cytoskeleton^58^, plant nuclei move rapidly and farther along the actin filament cytoskeleton^59^. A similar scenario was observed in sperm nuclear migration. Studies on male pronuclear migration in animal cells have demonstrated that microtubules are the preferred tracks for male pronuclear movement^60^, while it was recently reported that microtubules are dispensable for migration of male and female gamete nuclei of *Arabidopsis* and rice, where the presence of intact F-actin cables is necessary^9^,^27^,^28^. Our observations also indicated that actin filaments and not microtubules act as exclusive tracks for sperm nuclear migration in both the fertilized egg cell and central cell. Both the monocotyledons maize and rice and dicocotyledons tobacco and *Arabidopsis* showed similar mechanisms, indicating that actin but not microtubule-dependent sperm nuclear migration is conserved in flowering plants. A actin-based sperm cell nuclear migration was regarded as the compensation of complete loss of microtubule–centrosome-dependent processes in flowering plants^9^.

### WASP-Arp2/3 complex signal cascade might be involved in sperm nuclear migration in tobacco and maize

In plants, the involvement of myosin motors has been reported in the actin based movement of mitochondrial^43^, peroxisomes^61^,^62^, and the Golgi apparatus^63^. The intracellular trafficking of vesicles and organelles in pollen tubes mainly depend on actin and myosin motors^37^,^40^,^64^,^65^. Moreover, it was reported that myosin XI-i functions in nuclear movement in roots, indicating that the nuclear movement depends on actin-myosin in *Arabidopsis*^66^. These reports implied that sperm nuclear movement might depend on myosin. However, it was shown that nuclear movement in growing pollen tubes was not affected in the mutant of myosin XI-I^67^, suggesting that the male germ line nuclear movement depends on different proteins. Although it was reported that the dynamic F-actin movement in the central cell is myosin dependent^9^, the authors did not show whether sperm nuclear migration was affected under myosin inhibitor treatment. In our experiments, mitochondrial movement was dependent on myosin since treatment of GFP-tagged mitochondria with 2 mM BDM inhibited their movement. However, sperm nuclear migration could not be inhibited by higher concentrations of BDM, indicating tobacco sperm nuclear migration is not myosin dependent.

Actin polymerization mediated by the WASP-Arp2/3 complex signal cascade is present in a wide variety of cellular processes, in addition to driving actin-based motility of intracellular pathogens^68^,^69^,^70^,^71^,^72^,^73^. The bacterium Listeria moves along the host cell’s actin microfilaments after infection, and the Arp2/3 complex is the major factor in the nucleation of actin assembly of the actin comet tail in motile bacteria^74^,^75^.

The Arp2/3 complex, originally isolated from *Acanthamoeba*^76^, is thought to be highly conserved in all eukaryotes^77^,^78^. In higher plants, homologues of the Arp2/3 complex was found exit in *Arabidopsis*, tobacco and maize^79^,^80^,^81^,^82^,^83^. Recent studies have emphasized WASP and Arp2/3 complex play a central role in plant deveopment^64^,^67^,^83^,^84^,^85^,^86^,^87^,^88^,^89^,^90^,^91^. Our results showed that WASP and Arp2/3 complex are involved in sperm nuclear migration in maize and tobacco since sperm nuclear migration was disrupted both upon wiskostatin and CB treatment. However, a discrepancy between the two drug treatments was observed as wiskostatin applications did not induce the breakdown of cytoplasmic strands in primary endosperms, which instead occurred in CB treated cells. Our results indicated wiskostatin treatment inhibits sperm nuclear migration by suppressing the activity of Arp2/3 complex and not by disrupting the cytoplasmic strands connecting sperm nucleus and female nuclei. We suggest that upon membrane fusion sperm cells may act in an invasive fashion, such as that of Listeria, to recruit actin and other cytoskeleton proteins in female cells to activate actin assembly required for migration in tobacco and maize.

The mechanism of WASP-Arp2/3 complex signal cascade involved in sperm nuclear migration does not appear to be conserved in plants, because no obvious reproductive phenotype was reported in WASP or Arp2/3 complex *Arabidopsis* mutants^64^,^67^,^83^,^88^,^89^,^90^. Our supplementary data also showed that the female gametophyte and embryo development in *arp2arp3* double mutant are similar to those observed in wild type, indicating that sperm nuclear migration depends on proteins other than the WASP or the Arp2/3 complex in *Arabidopsis*. It was recently reported that dynamic F-actin activated by ROP8 is necessary for the migration of male and female gamete nuclei of *Arabidopsis*^28^, while the molecular downstream ROP8 is not clear. Since WASP or Arp2/3 complex do not function in sperm nuclear migration in *Arabidopsis*, other protein such as formins may be involved in the process.

Although there is no evidence to support the hypothesis that WASP or the Arp2/3 complex plays a role in sperm nuclear migration in *Arabidopsis*, our findings strongly suggest the WASP-Arp2/3 complex signal cascade drives sperm nuclear migration in tobacco and maize.

### Actin-dependent sperm nucleus migration is not a gamete specific mechanism

Our work indicates that in both tobacco and maize sperm nucleus migration in the egg cell is an actin-dependent process. This appears to be a common and conserved mechanism in fertilization in these two species. This observation led to the hypothesis that this mechanism could be a conserved mechanism for nucleus migration in all cell types. Cell fusion, in addition to fertilization, has been observed in somatic cell fusion for somatic cell hybridization^92^,^93^. However, the mechanism regulating the migration of these somatic cell nuclei is not clear. More interestingly, is the mechanism regulating sperm nucleus migration in egg cell gamete specific? Our observations on sperm nucleus migration and treatments with inhibitors indicate that the process is not likely to be gamete specific, but rather by a conserved mechanism for sperm nuclear migration within cells. Sperm nucleus migration may be regulated, as is motion of intracellular pathogens or other actin-dependent organelles, and thus may follow a general and conserved molecular mechanism. This notion may provide new clue for the investigation of the mechanism of fertilization by employing more convenient materials.

## Additional Information

**How to cite this article:** Peng, X. *et al*. The WASP-Arp2/3 complex signal cascade is involved in actin-dependent sperm nuclei migration during double fertilization in tobacco and maize. *Sci. Rep.* **7**, 43161; doi: 10.1038/srep43161 (2017).

**Publisher's note:** Springer Nature remains neutral with regard to jurisdictional claims in published maps and institutional affiliations.

## Supplementary Material

Supplementary Information

Supplementary Movie 1

Supplementary Movie 2

## Figures and Tables

**Table 1:** Effects of different inhibitors on sperm nuclear migration.

Fusion cell pair	Inhibitors	Sperm nuclear migration stop	Number
Sperm cell and egg cell of maize	colchicine	no	8
	cytochalasin B	yes	12
	LatA	yes	8
	BDM	no	8
	wiskostatin	yes	7
Sperm cell and egg cell of tobacco	colchicine	no	7
	cytochalasin B	yes	7
	BDM	no	6
	wiskostatin	yes	5
Sperm cell and central cell of tobacco	colchicine	no	5
	cytochalasin B	yes	8
	BDM	no	11
	wiskostatin	yes	5
Sperm cell and ovule protoplast of maize	colchicine	no	5
	cytochalasin B	yes	5
	BDM	no	5
	wiskostatin	yes	5
