# Supplementary material for: The WASP-Arp2/3 complex signal cascade is involved in actin-dependent sperm nuclei migration during double fertilization in tobacco and maize
Source: Sci Rep. 2017 Feb 22;7:43161. doi: 10.1038/srep43161 (PMC5320560; doi:10.1038/srep43161)
Supplement: Supplementary Information [file srep43161-s1.pdf]

The WASP-Arp2/3 complex signal cascade is involved in actin-dependent sperm nuclei migration during double fertilization in tobacco and maize

Xiongbo Peng[*], Tingting Yan, Mengxiang Sun

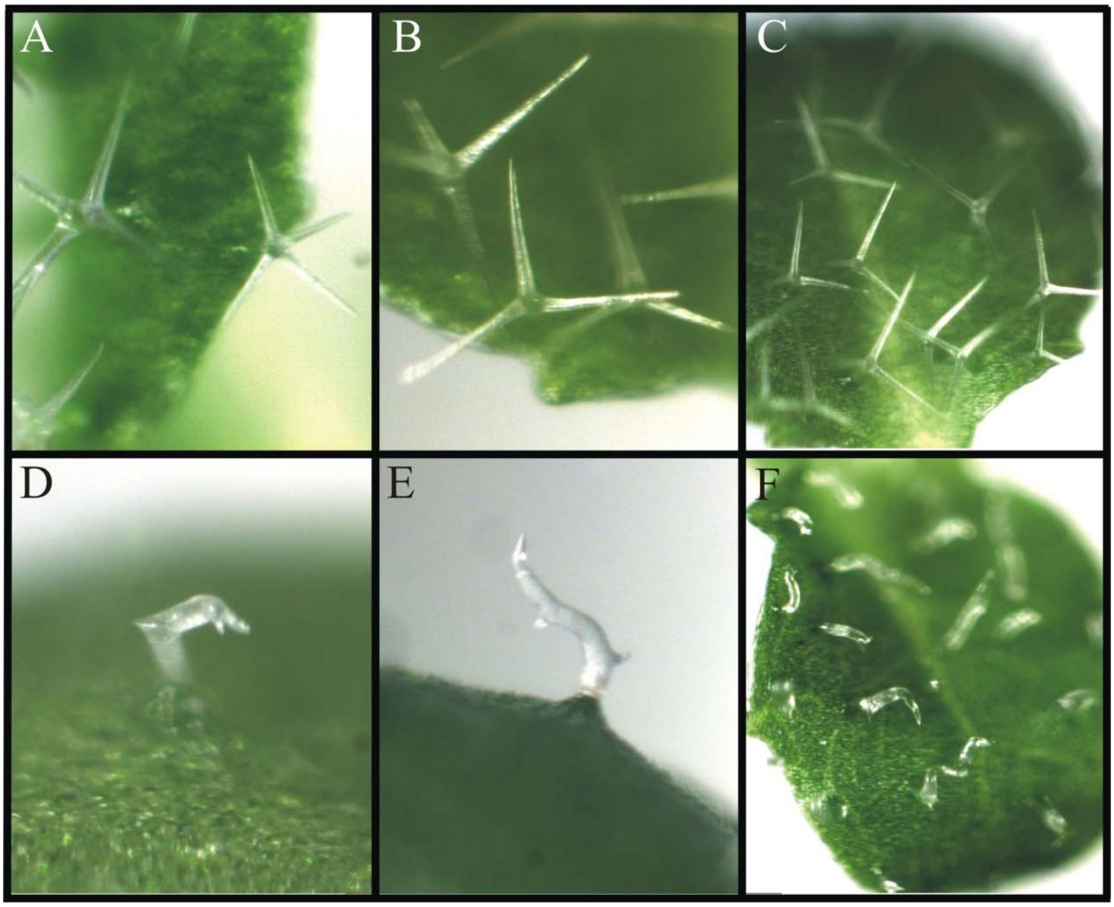

Supp Fig1 Arp2/3 complex mutant plants show abnormal trichome.
A wide type; B *arp2*/+ mutant; C *arp3*/+ mutant;
D *arp2/arp2* mutant; E *arp3/arp3* mutant; F *arp2arp3double* mutant.

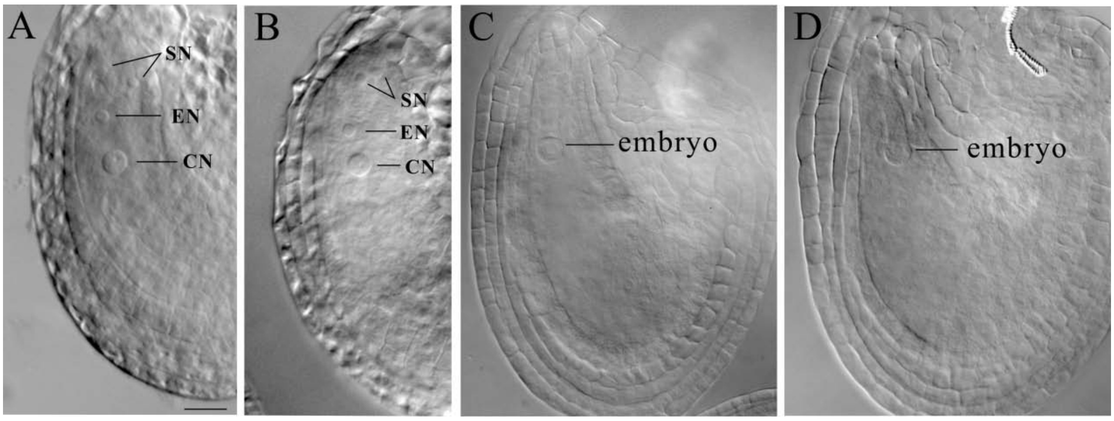

Supp Fig2 ***arp2arp3*** **double mutant plants show normal female gametophyte and embryo development.**

A wide type female gametophyte; B *arp2arp3* mutant female gametophyte; C wide type ovule one day after fertilization; D *arp2arp3* mutant ovule one day after fertilization. SN: synergid cell nucleus; EN: egg cell nucleus; CN: central cell nucleus.
